# Indigenous Influence and Engagement in Mining Permitting in British Columbia, Canada: Lessons for Sweden and Norway?

**DOI:** 10.1007/s00267-021-01536-0

**Published:** 2021-10-26

**Authors:** Christina Allard, Deborah Curran

**Affiliations:** 1 Division of Social Sciences/Law Unit, Luleå University of Technology, Luleå, Sweden; 2 Faculty of Law and School of Environmental Studies, University of Victoria, Victoria, British Columbia Canada

**Keywords:** Mining, Indigenous peoples, Sami, British Columbia, Sweden, Norway

## Abstract

Mine developments in Indigenous territories risk disrupting Indigenous cultures and their economies, including spiraling already high levels of conflict. This is the situation in Canada, Sweden, and Norway, as elsewhere, and is fostered by current state legal framework that reflect historical trajectories, although circumstances are gradually changing. Promising institutional changes have taken place in British Columbia (BC), Canada, with respect to new legislative reforms. Notably, new legislation from 2019 intends to implement the United Nations Declaration on the Rights of Indigenous Peoples (UNDRIP) in the province, by promoting consent-based and collaborative decision-making mechanisms. New environmental assessment legislation is another example; this legislation includes early engagement, collaborative decision-making, and Indigenous-led assessments. The article’s aim is, first, to analyze how Indigenous communities can influence and engage in the mining permitting system of BC, and, secondly, to highlight the positive features of the BC system using a comparative lens to identify opportunities for Sweden and Norway regarding mining permitting and Indigenous rights. Applying a legal-scientific and comparative analysis, the article analyzes traditional legal sources. The article concludes that the strong points that the BC regime could offer the two Nordic countries are: the concept of reconciliation, incorporation of UNDRIP, the spectrum of consultation and engagement approaches, and the structure of environmental assessments. All three jurisdictions, however, struggle with balancing mine developments and securing Indigenous authority and influence over land uses in their traditional territories.

## Introduction

The rush for minerals and new mine developments, accentuated by climate change and a “green transition” of our energy production, often leads to complex decision-making processes and local resistance (Addison and Roe 2018; Beland Lindahl et al. 2018). Mine developments in Indigenous territories are even more complex with Indigenous communities^1^ asserting rights and mandating a larger role in managing their traditional lands and the resources located there (Roy 2019; Muir and Booth 2012; O’Faircheallaigh 2010; Koivurova et al. 2015). In this context, in countries such as Canada, Sweden, and Norway, a growing demand for minerals at the global level with an increase in forthcoming decades in exploration and mining initiatives, mineral development risks severely disrupting Indigenous cultures and economies, and augmenting the already high level of conflict that is mediated by a state-led institutional landscape whose legal infrastructure and processes directly approve mining projects.

One example of this increasingly complex interaction is the conflicts between Indigenous authority and the state mining regime in British Columbia (BC), Canada (The Guardian 2021). On the one hand, most First Nations in BC have never ceded or surrendered their traditional territories and many of the long-standing disputes between the Province of BC and First Nations relates to the location or operation of a mine (Bennett 2020; The Canadian Press 2017). All lands in BC form part of the traditional territories of Indigenous communities, and mining law makes almost all lands in the province available for mining, except for the small areas of “prohibited access” lands, such as Class A parks. One could say that today’s modern Indigenous rights landscape collides with many parts of the mining regime that reflect a 19th-century approach that has continued to allow significant ecological impacts to Indigenous communities’ lands and waters.

On the other hand, in the context of Indigenous authority and working towards reconciliation between state and Indigenous societies, Canada is commonly considered a frontrunner jurisdiction, with clear constitutional recognition of aboriginal rights (*Constitution Act, 1982* s. 35; Macklem 2001: 48–49), and new federal and BC environmental assessment laws that acknowledge Indigenous organizations and participation. Specifically, the BC *Environmental Assessment Act, 2018* (*EA Act)* requires the Environmental Assessment Office to support reconciliation with Indigenous peoples by recognizing the inherent jurisdiction of Indigenous peoples and supporting the implementation of the United Nations Declaration on the Rights of Indigenous Peoples (UNDRIP). In fact, BC is the first Canadian province to apply UNDRIP to its own laws.

In 2018 a clear shift occurred when the Province of BC provided strong direction to the public service on renewing relationships with Indigenous people through government-to-government relationships based on recognition of Indigenous jurisdiction and laws where “[w]e will recognize success when we know Indigenous peoples believe themselves to be self-determining, self-governing, self-sufficient and can practice their Indigenous cultural traditions and customs as an important and respected part of B.C. society” (Province of BC 2018: 1). The intent of this direction is to change the orientation of the state government towards Indigenous peoples in line with Canada’s constitutionally acknowledged aboriginal rights and UNDRIP. One of the key implementation tools for this shift is the *Declaration on the Rights of Indigenous Peoples Act, 2019* (BC *DRIPA*) enacted in November 2019 to align BC laws with UNDRIP, which the Truth and Reconciliation Commission of Canada called for as a framework for reconciliation (TRC 2015). The federal government has also recently enacted the *United National Declaration on the Rights of Indigenous Peoples Act, 2021* with similar purpose.

With this backdrop of considerable state recognition of Indigenous authority, this article aims first to explain mining-related laws of BC for an international audience, with a particular focus on how Indigenous communities can influence and engage in the decision-making processes related to mining. Secondly, it aims to highlight the positive features of the BC system using a comparative lens to identify opportunities for Sweden and Norway with respect to their regulatory systems for mining and Indigenous rights.^2^ In Sweden and Norway the Indigenous Sami, performing their traditional livelihoods of reindeer herding, hunting, and fishing in vast northern areas, have, especially over the past decade, been faced with increased pressure by other land uses, not the least by mining projects (Raitio et al. 2020; Nygaard 2016).

Methodologically, this article presents a legal-scientific and textual analysis of key legal sources, including legislation, case law, and legal literature as well as other scholarly literature and public documents. In addition, a legal-comparative lens (e.g., Zweigert and Hein Kötz (2011): 4–5) is utilized for assessing the extent to which the BC legal system facilitates Indigenous authority, influence, and engagement, and draws conclusions that may prove instructive for Swedish and Norwegian mineral-related laws and permitting processes regarding Sami rights.

After this introduction, the article is structured as follows. First, we provide context on Indigenous communities’ relationship to the state, rights, and controversies, and the impacts of mine developments. Second, we examine the BC laws relating to the development of large mineral mines and focus especially on which permit processes Indigenous communities can influence and participate in. The section ends with an analysis of problematic features in the BC regulatory system as well as the potential for change because of new legislation and government-to-government agreements. Third, we describe the status of Sami rights in both Sweden and Norway and highlight the weak engagement by the state with the Sami in mine permitting processes. Fourth, the article explores lessons for Sweden and Norway from a comparative standpoint, and lastly, we set out our conclusions related to Indigenous authority, influence, and engagement for mine developments.

## Context: Mine Developments, Indigenous Communities, and Evolving Practices in BC

Mining legislation is among the oldest state laws in BC and has, historically and up to today, played a central role in attracting non-Indigenous settlers and investment. This westernmost province in Canada^3^ is rich in mineral resources and it was gold that attracted many settlers as the promise of gold saw miners spreading throughout BC in waves. Like the gold rushes in California and Yukon, BC experienced immigration and settlement relating to mining for gold via the Fraser Canyon and Cariboo gold rushes prior to the mid-1860s (Barman 1996).

After the initial frenzy for gold, the development of mines steadily increased. Coal was the other mineral commodity produced from around the 1860s, with mines yielding silver, copper, lead, and zinc also established before the turn of the 20th century. By 1960 iron and molybdenum joined the output (Barman 1996: Table 20). These historic and contemporary mining activities have created environmental impacts that are still being addressed today.^4^ While the contemporary landscape of Indigenous authority and mining in BC is evolving rapidly due to both Indigenous and state processes, the mining industry’s interest remains strong. BC advertises itself as “the ideal business environment for extractive industries” and is home to the largest concentration of mineral exploration and mining professionals in the world, with over 700 having Vancouver as their global operations base (Province of BC no date-b). With 72 major developed mine sites across BC and 16 environmental assessment processes underway, the estimated value of production in 2019 was $8.8 billion, with $423 million in exploration spending in 2020 (Province of BC no date-c). Mining exports increased by 139 percent between 2013 and 2016 (Province of BC no date-b).

Mineral exploration and mine development continue to be controversial. Most recently, a coalition of 200 Indigenous, business, and environmental organizations are opposing Imperial Metals’ application for an exploration permit to drill for gold near the Canada-US border in the headwaters of the Skagit River (The Guardian 2021). Formally, through their elected leadership, some Indigenous communities support some mining, such as the Tahltan Central Government whose traditional territory is subject to 41 percent of mineral exploration in the province (no date; Fidler 2010; Business Wire 2021). Other communities, including the Tsilhqot’in National Government, continue to oppose mining exploration and proposals in their traditional territories (Canadian Press 2019; Lavoie 2019).

The map of Indigenous communities is complex in BC; 34 Indigenous language groups (First Peoples Cultural Council no date; University of BC Museum of Anthropology 1996) and 203 distinct First Nations that represent Indigenous communities (Indigenous Services Canada 2019). The province was one of the latest areas of Canada to be colonized, and joined the Canadian federation in 1871. Whereas the federal government signed treaties throughout the western part of Canada between 1871 and 1921, except for some pre-confederation treaties on Vancouver Island and a post-confederation treaty in the northeast of the province (Godlewska and Webber 2007), it did not enter into treaty relationships with most of the Indigenous communities in BC (Foster and Grove 2008). Throughout Canada and in BC, the federal government restricted Indigenous communities to a very small portion of each community’s traditional territory as Indian reserves on which they could live. The rest of the provincial lands and waters were open for settlement by non-Indigenous people and resource development (Harris 2008).

For most of the 20th century the federal and provincial governments ignored Indigenous claims and authority, including when permitting natural resource extraction (Foster et al. 2007; Hoogeveen 2015). Having responsibility for mines and minerals (*Constitution Act, 1867*), the colonial governments and subsequently the Province of BC established a mineral tenure regime that permitted mineral claims and mining throughout Indigenous communities’ traditional territories as “waste lands of the Crown” (*Gold Fields Act, 1859*; *Mineral Act, 1936*; Hoogeveen 2018). Across the incredibly diverse socio-ecological landscape where distinct Indigenous legal orders organized Indigenous societies in relation to their environments (Asch et al. 2018; Napoleon 2009), state laws permitted extractive activities irrespective of Indigenous laws and legal processes. Thus, there is an undercurrent of Indigenous claims and petitions for authority over their traditional territories (Foster 2007) to all mineral extraction activities.

Although the Supreme Court of Canada had already acknowledged in 1973 that aboriginal land rights and title still exist in Canada (*Calder* 1973), it was not until 1982 that the federal government amended the constitution to “recognize and affirm” aboriginal and treaty rights (*Constitution Act, 1982*, s. 35). Subsequent court decisions defined those aboriginal rights in BC as rights to carry out practices, customs, and traditions integral to the Indigenous society (*R v* *Van der Peet* 1996), typically including the rights to hunt, fish, gather, and carry out cultural practices for food, social and ceremonial purposes (*R v* *Sparrow* 1990). Within this framework the Supreme Court of Canada ruled that the primary purpose of section 35 of the *Constitution Act, 1982* was to direct aboriginal rights “…towards the reconciliation of the pre-existence of aboriginal societies with the sovereignty of the Crown” (*Van der Peet*: para 31*)*. True reconciliation, therefore, would take into account both the view of Indigenous peoples and the common law or state governments (*Van der Peet*: para 49–50*)*. This view of reconciliation allows state governments to infringe aboriginal rights within a traditional territory where “limits placed on those rights are, where the objectives furthered by those limits are of sufficient importance to the broader community as a whole, *equally* a necessary part of that reconciliation” (*Gladstone;* para 73 emphasis in original*)*. Infringement of aboriginal rights is justified for the purpose of the development of society, which includes, for instance, mineral extraction and the development of infrastructure (*R v* *Sparrow* 1990; *Delgamuukw* 1997; *Tsilhqot’in Nation v British Columbia* (2014)). However, in order to infringe an aboriginal right, the state is required to consult and accommodate the affected Indigenous community when a proposed activity may have an impact (*Haida Nation* 2004; *Taku River Tlingit* *v British Columbia* 2004). This consultation and accommodation interaction has been the activity that encompasses much of the relations between First Nations and the state.

A key question for courts over the past 20 years has concerned whether the state has adequately consulted and accommodated an Indigenous community when a decision by the state is going to have an impact on a community’s aboriginal rights. This duty to consult extends beyond decisions about land and the environment to all aspects of Indigenous cultural practices, which requires the state to consult widely on many decisions. Depending on how strong the relationship (and the claimed right) is between an Indigenous community and the place where a proposed activity will occur within a traditional territory, there is a spectrum of consultation possibilities (*Haida Nation v British Columbia Minister of Forests*, 3 S.C.R. 511 (2004); *Taku River Tlingit* 2004). These range from mere information sharing to ongoing meetings and discussions over a long time period with the proponent altering the proposed project or the state refusing to approve the project (see also Newman, 2014: 90–91, 104–5). A majority of this case law concludes that the state adequately fulfilled its duty to consult (e.g., *Taku River Tlingit* 2004; *Halalt First Nation v British* *Columbia* (2012)). Within the aboriginal rights context there is no definitive standard by which a court will uphold an Indigenous community’s view that, for example, logging or the establishment of a mine in a sacred area is inappropriate (*Tsilhqot’in Nation v British Columbia* (2014); *Ktunaxa Nation* (2017)). First Nations have no ability to veto a proposed development or the expansion of existing operations. Therefore, the duty to consult and accommodate does not reflect a standard of consent as anticipated by UNDRIP, and largely permits mine development to continue across much of BC.^5^

The current treaty process is also affecting the mining regime in BC. Since 1997 Indigenous communities have been negotiating modern treaties with the federal and provincial governments (BC Treaty Commission 2020). Most First Nations have entered into the treaty process but many of them have since stopped actively negotiating given the narrow negotiating mandates of the state governments (Egan 2012). As a result, there are few signed modern treaties in the province. The few that do exist provide the signatory Indigenous communities with some control only over a small portion of their traditional territory with continued state authority over essential resources and processes such as water and environmental assessment (e.g., Maa-nulth First Nations, Government of Canada, Province of British Columbia (2009)). Some Indigenous communities have gained control over some subsurface resources through modern treaties but only those under the treaty lands controlled by the First Nation (e.g., Maa-nulth First Nations, Government of Canada, Province of British Columbia (2009) clause 4.1.1).

More recently, Indigenous communities are turning to revitalizing their own laws and legal processes, and are using these legal orders in part to respond to state approval processes for natural resource extraction. For example, in 2016 the Stk’emlupsemc te Secwepemc Nation convened its own community process pursuant to Secwepemc laws, traditions, customs, and land tenure systems to assess whether members would give their free, prior, and informed consent to a proposed mine in the community (see further below). Concluding that they would not give consent for the development of the mine, members of the Nation’s elected council noted that the affected area, called Pipsell, is a cultural keystone place with which the community has a spiritual connection. The irreversible impacts of the proposed mine on the environment and cultural heritage were simply unacceptable (Stk’emlupsemc te Secwepemc Nation 2017).

The revitalization of Indigenous legal processes is occurring alongside new provincial legislation that implicates land and resources, and offers potential for creating new models for Indigenous-state relations. BC is the first jurisdiction in Canada and one of the first in the world to establish a legal framework for the implementation of UNDRIP. The purpose of the BC *Declaration on the Rights of Indigenous Peoples Act, 2019*^6^ is to affirm the application of UNDRIP vis-à-vis the laws of BC and thereby contribute to its implementation (s. 2). Equally important, section 3 of the BC *DRIPA* states that “[i]n consultation and cooperation with the Indigenous peoples in British Columbia, the government must take all measures necessary to ensure the laws of British Columbia are consistent with the Declaration.” This means that the various mining laws, as well as other laws, must be made consistent with UNDRIP, which anticipates future reforms of legislation to align various provisions with the ideals enshrined in UNDRIP. Moreover, the new BC *DRIPA* enables agreements between the provincial government and an Indigenous governing body^7^ (s. 6), and explicitly allows the co-exercise of or consent for the exercise of statutory powers of decision (ss. 1, 7). Importantly, it is this final element that foreshadows collaborative governance arrangements between the provincial and Indigenous governments that include consent-based processes.

It is through the BC *DRIPA*’s collaborative decision-making mechanisms (ss. 6-7) that the BC government may significantly advance the goals of UNDRIP beyond what most jurisdictions in the world have committed. It permits the Province of BC to delegate decisions to Indigenous governing bodies, including agreement on consent-based processes before approving projects such as mine developments.

It is, however, important to note that the BC *DRIPA* is still very new, as are the provisions of the *EA Act* that address Indigenous peoples. The existing but evolving regulatory regime for mining in BC still makes available most of the landscape in the province, much of which is not subject to specific treaty rights and for which Indigenous nations have proven or claim aboriginal rights and title. While these new Indigenous-led and focused statutory provisions have significant potential for Indigenous nations to influence mining, they do not yet have meaningful application in the context of the existing mineral exploration, permitting, and operating regime.

## The Mining Permitting Process and Engagement with First Nations in BC

### The Basis for Engagement with First Nations

There are three distinct sources of legal entitlement to participate or have the state government engage with First Nations: (i) A constitutionally recognized and affirmed aboriginal or treaty right; (ii) According to provincial environmental assessment and other statutes that mandate consultation or collaborative decision-making; or (iii) Pursuant to unique government-to-government (also called reconciliation) agreements between First Nations and a state government. In the provincial mine permitting process, the two most prevalent engagements—consultation and via statute—take place only after mineral claims and some exploration have occurred. Considerable controversy can occur before mine development, but, as explained below, the provincial regime has not yet modernized to address that temporal and spatial weakness.

First, under the constitutional duty, the general rule is that whenever provincial government staff or elected officials make a decision that may have an impact on an Indigenous community’s lands or waters, and thus their aboriginal or treaty rights, they must consult with the potentially affected First Nation. This duty to consult and accommodate is ongoing and is triggered again whenever a state government decision may have an impact. A First Nation can challenge a state government decision alleging inadequate consultation, as well as on any other legal basis. Second, statutory duties arising from laws such as the new *EA Act* arise only in the specific context set out by that law (see further below). In the case of the *EA Act*, the ability to participate and be involved in decision-making endures only for the duration of the environmental assessment. There is no ongoing duty to consult.

Third, there are many government-to-government agreements between First Nations and the Province of BC that spell out unique consultation processes related to specific types of decisions. These government-to-government agreements are typically between two and ten years in length, and often reflect a more comprehensive agreement about how specified activities such as forestry will occur within the signatory First Nation’s traditional territory. Mining laws themselves do not provide any opportunity for Indigenous communities to participate in decision making. It is the legal superstructure of constitutional aboriginal rights, environmental assessment, government-to-government agreements, and the new BC *DRIPA* that create possibilities for deep engagement and joint decision making in BC.

### Overview of the Mining Law Regime

Depending on the type of mineral and the size of the mine, mine developments in BC follow different approval processes. The lifecycle of larger mines, called major mines, involves five components: (1) Mineral tenure or claims; (2) Exploration; (3) Development; (4) Operation; and (5) Closure and reclamation. This article focuses on the legal requirements for phases 1 to 3 (mine development) in the context of Indigenous communities’ ability to influence and engage in decision making. In summary, while the aboriginal rights framework requires consultation with First Nations, the fundamental actions of mineral staking and exploration occur largely before any interaction between Indigenous communities, the Provincial Government, and mine proponents. Therefore, the mine tenure system, characterized as “free entry”, precludes the fundamental conversation about whether any activity is appropriate in a specific location before proponents secure legal rights under state mining law.

The mining law regime in BC is, overall, well-critiqued. Several reports relating to mining and mining law in the past five years have pointed to regulatory weaknesses that threaten the public interest. For example, in 2016 the Auditor General^8^ of BC found that the regulatory compliance and enforcement activities of the provincial Ministry of Mines and Ministry of the Environment were inadequate. Noting that the Ministries’ behavior has increased environmental risk and limited the state government’s ability to protect the environment, the Auditor General recommended that the compliance and enforcement functions be removed from the Ministry of Mines which is also responsible for promoting mine development. Further, in 2020 a coalition of over 20 environmental, community, and Indigenous organizations released the BC Mining Law Reform platform setting forth consensus recommendations for updating all areas of the mining law regime, including creating meaningful consent-based relationships with Indigenous communities (BC Mining Law Reform Network 2020).

In addition, despite the recent enactment of the BC *DRIPA* and commitment to implementing amendments relating to UNDRIP, the BC provincial government has not yet comprehensively reformed mining laws to respond to Indigenous authority and the call for consent-based processes.

### The Right to Minerals

Despite the traditional rule in English common law that a fee simple title extends “from the center of the earth to the heavens above” (Newman 2018: 59, 64), Canadian law maintains a separation both between surface and subsurface rights and between minerals owned by the Crown (federal and provincial governments) and minerals owned by the property owner (ibid: 59–60). However, freehold mineral rights in Canada, and definitely in BC, are very much the exception rather than the rule (ibid: 64). A common denominator throughout Canada is the Crown ownership of mineral rights (Crown minerals), although the ownership of minerals today is complex and also coincides with legal recognition of Indigenous rights and increasing claims of Indigenous ownership of minerals (Newman, 2018: 59–60, 62). The claim of Crown ownership of most minerals throughout Canada rests either on the basis that the Crown owns the land or that the Crown has granted land to private landowners and at the time of granting it reserved mineral rights to the Crown (ibid: 59–60). In BC, the Provincial Government claims ownership to 94 percent of the landscape and, therefore, much of its claim to ownership of minerals stems from the ownership of land (Province of BC 2011).

With this system, any qualified proponent can normally secure the acquisition of Crown mineral rights through prospecting, which is the result of the historical “free entry” or “free miner” system that causes problems with respect to aboriginal and treaty rights (e.g., Drake 2015: 190–91); over time this system has taken different forms across Canada (Newman 2018: 75). As described, BC’s current mining legislation rests upon early colonial gold rush era laws (Barman 1996); the prospectors were given a right of “free entry” to most lands in the colony, including a system for them to acquire mineral rights by “staking a claim”.

### Three Phases of Mine Development

The first mining phase is mineral tenure or mineral claims. Under BC mining laws applicants can stake a claim and obtain a *mineral tenure* anywhere in the province, except for about 18 percent of the land base that is subject to designations as conservation areas, or protected heritage or cultural property (*Mineral Tenure Act*, 1996 ss. 11(2), 14(5), 17, 21). The provincial government *must* issue a “free miner certificate” upon application and fee payment (*Mineral Tenure Act*, 1996 ss. 7, 8) to a qualified individual or company. The holder of such certificate can then pay a fee and click on a map to register a claim for a *mineral title*^9^ (*Mineral Tenure Act* ss. 6.2, 6.3; *Mineral Tenure Act Regulation* 2016 s. 4; Stano & Lehrer 2013; Province of BC no date-d).

Significantly, the Supreme Court in Canada’s northern Yukon Territory ruled in 2011 that this type of online claim system triggers a duty to consult affected Indigenous communities (*Ross River Dene Council v Yukon* 2011), but the BC system still permits the registration of a claim without consultation with the affected Indigenous community or consideration of the sensitivity of watersheds or other ecological or cultural conditions (Hoogeveen 2018). Moreover, the mineral claim process is exempt from land use plans and zoning regulations, and even applies on private land (*Mineral Tenure Act* ss. 11, 19).

Under BC’s “free entry” system the claim holder is entitled to the minerals located vertically downwards from the boundaries of the claim (*Mineral Tenure Act* s. 28), which permits the use and occupation of the claim area for the purposes of exploration and to develop the mineral resources (*Mineral Tenure Act Regulation* s. 4(9)). In fact, exploration and development activities must occur annually, and be registered or a payment made in lieu of activity, to maintain the claim year-over-year (*Mineral Tenure Act Regulation* ss. 7(1), 8, 10; *Mineral Tenure Act* ss. 29, 33.1).

Following the *Mineral Tenure Act* and regulations, the use of land includes “the treatment of ore and concentrates, and all operations related to the exploration and development or production of minerals (…) and the business of mining” (s. 14). This permits the claim holder to develop up to a specific volume of minerals, which is 1000 tonnes of ore per year or a 10,000-tonne bulk sample once every five years (*Mineral Tenure Act Regulation* s. 17). Hence, a mineral tenure allows exploration activities, which relate to the second mining phase, and mining operations up to a certain level. According to the definition in the *Mineral Tenure Act Regulation* “exploration and development” include both physical and technical exploration and development (s. 1).

Production beyond the stipulated volumes mentioned above requires the claim holder to convert the claim into a *mineral lease*, which again, the Province of BC *must* issue for a 30 year period if the claim holder pays a fee, surveys the area of the mineral claim, and provides specific public notice of the lease application (*Mineral Tenure Act* s. 42). The Province will normally approve surface rights giving access to the claim area for mining activity, if needed (*Mineral Tenure Act* s 15). While notice must be given to owners of surface areas of land or leaseholders of public land where mining activities will occur (*Mineral Tenure Act* s. 19(1)), no such requirement exists for notice to Indigenous communities who claim or have recognized aboriginal rights in the claim area.

All of this can occur before an environmental assessment is required, and these exploration and other activities can be controversial. For example, in 2019 the Tsilhqot’in Nation challenged an exploratory drilling program (a Notice of Work permit, see below) and sought a court injunction to prohibit the mining company (Taseko) from carrying out the activities (*Taseko Mines Limited v*. *Tsilhqot’in National Government* 2019). In awarding the injunction to the Tsilhqot’in Nation (leave to appeal denied), the BC Supreme Court found that the Tsilhqot’in community stood to suffer greater irreparable harm than the company (at para. 129). The court also weighed the public interest and found that it tipped in the Tsilhqot’in Nation’s favor due to the imperative of reconciliation between Indigenous nations and the state (at para. 131).

The third mining cycle phase relates more directly to the development phase, or specifically the approvals necessary for operating a mine, which is also the first formal requirement for consultation with First Nations and their engagement with mineral development. Operating a mine includes mechanical disturbance, excavation, or drilling of the ground for exploration activities, as well as other cleared areas used in servicing a mine (*Mines Act*, *1996* s. 1). The *Mines Act* applies to all mines and during all phases of a mining cycle (exploration, development, construction, production, closure, reclamation and abandonment) (s. 2). Under the *Mines Act* approval for a mine is required before commencing work in or around a mine (s. 10), with the applicant needing to file a plan outlining the details of the proposed work and a program that considers the conservation and protection of cultural heritage resources, land and watercourses (s. 10(1)).

The operation of a mine involves multiple different types of approvals, including decision makers in different Ministries and various laws. Permits are needed for the exploration activities under the *Mines Act*, waste management under the *Environmental Management Act*, permits to cut trees pursuant to forestry legislation, road use permits, and authorizations for changes in and about streams and for the diversion of surface and groundwater pursuant to the *Water Sustainability Act*.

For smaller-scale industrial mines, proponents submit applications for what is known as a “Notice of Work” or regional mines through FrontCounter BC, the single administrative portal for permit applications dealing with land, water, and natural resources. A regional Mine Development Review Committee or staff conducts a technical review and refers the application to other government agencies and Indigenous communities for input, after which the applicant can provide a response and the statutory decision-maker renders a decision (Province of BC 2020). For major mines that will be producing a mineral or coal, this permitting process is coordinated across the province by the Major Mines Permitting Office, which closely coordinates with the Environmental Assessment Office (BC Major Mines Office no date).^10^ The Office works directly with proponents, First Nations, and government technical advisors to coordinate multi-agency regulatory permits (ibid).

Consultation with Indigenous communities is ongoing and occurs at different stages of the permitting process. For example, state governments and the proponent will engage with First Nations about the activity or project itself and then as each permit is drafted and assessed. Considered a best practice, the Province of BC encourages applicants to engage with Indigenous communities prior to submitting applications for approvals (Province of BC 2020). While the Province has a duty to consult Indigenous communities on potential infringement of their aboriginal and treaty rights, it may delegate some procedural aspects of consultation to project proponents (Province of BC no date-d). Consultation can include supplying information about the proposed project or activity, providing a reasonable opportunity for Indigenous organizations to review and provide comments on the proposal, and considering input provided and modifying project plans where feasible. Any modifications, called accommodation, arise from the constitutional aboriginal rights framework’s duty to consult and accommodate potential negative effects or infringement of aboriginal or treaty rights caused by the mining project.

### Engagement and Environmental Assessment

Developing a mineral claim further requires an environmental assessment (EA) for major mines or modifications of existing mines. This happens normally after a mineral lease is secured, some exploration has occurred, and in conjunction with the third mining cycle phase of development. At this point the Major Mines Office (MMO) coordinates applications and permits, as well as closely liaises with the EA Office. As noted initially in this part, the requirement for state governments to engage with First Nations stems from three separate types of authority: the constitutional duty to consult and accommodate, specific legislation such as the *EA Act*, and by government-to-government agreement. For environmental assessment we focus on the second, statutorily-based, authority for engagement.

Under BC law, the threshold for requiring an EA for new mines is 75,000 tonnes per year of mineral ore under the *Reviewable Projects Regulation, 2019* (ss. 9, 10).^11^ This relatively high threshold for engaging the EA process means that many mining activities are not subject to comprehensive review and exist only under the various permitting processes for land, water, and waste management. Approval of the project via the EA application and the EA report by the responsible minister under section 29(4) of the *EA Act* results in an “environmental assessment certificate” issued to the proponent. The principal assessment objectives are to promote sustainability and reconciliation with Indigenous peoples, as stated in section 2(2) of the *EA Act*. Moreover, the EA has a broad scope for assessment and directs that the process consider “the environmental, economic, social, cultural and health effects of assessed projects” (s. 2(2)(b)(i)(A)).

Under the new *EA Act* the purposes of the EA Office, the independent office of the provincial government responsible for administering the EA, includes the integration of Indigenous knowledge and supporting reconciliation with Indigenous peoples in BC by (s. 2(2)(b)(ii)):

(A) supporting the implementation of the United Nations Declaration on the Rights of Indigenous Peoples,
(B) recognizing the inherent jurisdiction of Indigenous nations and their right to participate in decision making in matters that would affect their rights, through representatives chosen by themselves,
(C) collaborating with Indigenous nations in relation to reviewable projects, consistent with the United Nations Declaration on the Rights of Indigenous Peoples, and
(D) acknowledging Indigenous peoples’ rights recognized and affirmed by section 35 of the *Constitution Act, 1982* in the course of assessments and decision making under the Act.

The *EA Act* is remarkable for its focus on early engagement, collaborative decision-making, Indigenous-led assessment, and some attention to consent. Indigenous nations receive specific consideration and status under the *EA Act* and are not simply part of the “general public”: Every assessment must address the effects of the project on Indigenous nations and their aboriginal and treaty rights (s. 25). When the initial project description is published an Indigenous nation may provide notice that they intend to participate in the assessment of the project (s. 14). The chief executive assessment officer *must* seek to achieve consensus on many decisions with those Indigenous nations obtaining status as participating Indigenous nations, including whether to proceed with the assessment (s. 16), process planning (s. 19) and preparing the draft assessment report (s. 28). This also includes the recommendation to the minister on whether the project “is consistent with the promotion of sustainability by protecting the environment and fostering a sound economy and the well-being of British Columbians and their communities” (s. 29). While timelines for responses are set out throughout, it is notable that state law dictates the Minister, ultimately, as the final decision-maker.

There is also provision for Indigenous-led assessment. A participating Indigenous nation can provide notice to the chief assessment officer that it intends to carry out an assessment of the potential effects of the project on the nation and its aboriginal or treaty rights, and the chief assessment officer must then specify the portion of the assessment to be undertaken by that Indigenous nation (s. 19(4)). While this provision has yet to be used in the mining context, in theory an Indigenous nation could undertake the entire EA process. The chief assessment officer may also decide on the costs to be paid by the proponent to the Indigenous nation to defray the Indigenous nation’s costs in participating in the assessment, also taking into account, for instance, the size and complexity of the project (s. 48).

Importantly, an Indigenous nation can express its decision to grant or deny consent at two stages of the process: (i) Whether to exempt the project from an assessment or terminate the project (s. 16); and (ii) The decision on the environmental assessment certificate (s. 29). If the recommendation to the minister about project approval contradicts the consent decision of an Indigenous nation, the minister *must* offer to meet the Indigenous nation before making a final decision (s. 29(5)). Even if this consent language does not amount to a veto for Indigenous nations, with the concept of veto generating considerable controversy (Imai 2017), it still signifies the importance of working towards a common solution through consensus-like procedures throughout the assessment process.^12^ Finally, an Indigenous nation can refer specified matters to a dispute resolution facilitator (s. 5).

### Longstanding Critiques and New Potential for Reconciliation

Between the three distinct state processes for engagement with Indigenous communities, most of the engagement is triggered by the first two – the duty to consult and accommodate required through Canada’s constitutional recognition of aboriginal and treaty rights, and engagement under specific statutes such as the *EA Act*. Both of these processes for consultations are activated by actual proposals for mining activities after mineral claiming and exploration have occurred. There are two structural problems with locating engagement at this stage.

First, the mine permitting and EA processes are incapable of assessing whether or not it is appropriate to locate a mine in a specific location. Mineral tenure is a fundamental issue that is often addressed for the first time at the EA stage and environmental assessment is not typically designed to examine if a particular site will ever be appropriate for a mine based on Indigenous knowledge, laws, and customs. Instead, environmental assessment is largely concerned with under what conditions it is acceptable to operate a mine in the proposed location. True influence by Indigenous peoples would address the threshold question of mineral tenure and location of extraction activities well before any mine permitting or an environmental assessment for a specific project arises.

The second structural problem is that once a mine is operating there are not necessarily ongoing state government decisions that trigger a duty to consult either based on aboriginal or treaty rights or by statute. The Indigenous communities that feel the impacts of operating, closed, or abandoned mines do not have transparent avenues through which their concerns can be lodged and addressed.

Therefore, with respect to the regulatory framework in BC, it is startling that the Provincial Government has no formal obligation to consult with First Nations or ability to deny specific authorizations under the mineral tenure regime at the time of claiming minerals. The Province of BC has no power under state law to pause to consult with affected Indigenous communities or to invite their participation if the mineral tenure holder follows the registration and access rules under the *Mineral Tenure Act* and *Regulation*. Nor does it have any discretion to balance different interests in the process of denying or awarding rights to minerals. At this stage there simply is no balancing or trade-offs of interests, and one could say that BC’s free entry system unjustifiably favors mining interests over public interests.

The 2016 Auditor General’s report noted the operational weaknesses of the mining regime, particularly in the lack of compliance and enforcement at all stages, in particular with EA. The Auditor General criticized the absence of a compliance and enforcement program in the EA Office, noting a failure to ensure adherence with EA certificates, carry out site inspections and issue penalties or cancel EA certificates. The Auditor General also found that the Ministry of Mines’ focus was predominantly on project applications and was under high risk of regulatory capture by the mining industry. The shrinking enforcement activities of the Ministry of Environment and the Ministry of Mines meant that they were unable to protect the province from environmental risk.

Superimposed onto these longstanding critiques, however, are the statutory possibilities offered by new legislation. The BC *DRIPA* offers new potential for agreements between the Provincial Government and Indigenous governing bodies that can create ongoing processes for considering *all* activities, including mineral staking and exploration, in an Indigenous traditional territory. The BC *DRIPA* goes beyond the constitutional framework of consultation that triggers specific decisions related to proposed projects and enables a more comprehensive and relational approach to decision-making. In addition, when major projects do get to the environmental assessment stage, the new *EA Act* creates several new avenues for Indigenous direction and participation as governments with unique status (i.e., not just another stakeholder). These mechanisms include the potential for early involvement, Indigenous-led assessments, and consent. Ultimately, however, it is important to note that the *EA Act* does not meet the minimum standard of UNDRIP for free, prior, and informed consent because projects can still go ahead when opposed by Indigenous communities – the Minister and other provincial officials retain ultimate authority to exempt projects from assessment, approve or reject projects, or make procedural decisions whether or not they have consensus from participating Indigenous nations. This weakness is repeated across the Canadian legal landscape (Scott 2020), and, overall, these new laws do not address the legacy of existing mines and mining impacts across BC.

While there is significant potential under the new *EA Act* and BC *DRIPA* for Indigenous communities to lead and be meaningfully involved in the siting and development of new mines, these provisions are untested. Although not an Indigenous-led assessment (under the new *EA Act*) the example of the Stk’emlupsemc te Secw*é*pemc Nation (SSN) demonstrates how Indigenous communities are using their authority to obtain agreement on how project assessment will occur, and to assess whether a mine proposal respects their Indigenous laws and cultural responsibilities (Boron and Markey 2020; Curran 2019).

In the case of the Ajax mine project, both the federal and provincial governments found that deep consultation was warranted for this project given the extent and location of the SSNs aboriginal rights (Impact Assessment Agency of Canada 2017). In 2016 the SSN and Province of BC signed a government-to-government agreement for a framework and collaboration plan for the EA and decision-making about the mine proposal in Secw*é*pemc traditional territory and, specifically, around Jacko Lake (Pipsell to SSN members). The agreement explicitly acknowledged SSN authority and laws, noting that the Stk’emlupsemc are the caretakers of Jacko Lake and area (Stk’emlupsemc te Secw*é*pemc Nation and Province of BC 2016, 7.d). As part of the agreement, the SSN created their own community-led assessment pursuant to their laws, customs, and traditions. SSN made a declaration of title to Pipsell (Jacko Lake and surroundings), a cultural keystone area with significant spiritual and historical importance to the SSN (Stk’emlupsemc te Secw*é*pemc Nation 2017).

The SSN concluded that they would not give their free, prior, and informed consent to the mine project due to its irreversible impact on Pipsell, in part because it contradicted the SSN’s land use objectives for Pipsell. Subsequently, through the EA process, the federal government refused to approve the mining project on the basis of evidence that the Ajax mine would likely cause significant adverse environmental effects that could not be justified in the circumstances (Impact Assessment Agency of Canada 2018). In this case, although the state governments asserted ultimate authority over the decision, the SSN was a collaborative partner in the process and evaluated the mine proposal according to their own traditions and laws as part of an expanded consultation framework.

The potential of the new statutory regime must be read in the broader context of state-Indigenous relations in BC where individuals or groups of Indigenous communities in ecologically renowned regions have succeeded in securing unique governance arrangements (Curran 2017). What BC appears to be moving towards, particularly in the context of BC *DRIPA*, is ongoing government-to-government processes for joint decision making for all or part of Indigenous traditional territories. There will not just be processes set up for an environmental assessment, but ongoing collaborative governance structures, as is seen in other parts of the province such as Haida Gwaii.

In the island archipelago of Haida Gwaii, on the Pacific coast, the Council of the Haida Nation and the Province of BC jointly manage forestry, protected areas, and cultural heritage. Through a reconciliation agreement signed by the parties as a government-to-government agreement, each party appoints an equal number of representatives to the Haida Gwaii Management Council that is responsible for establishing tree-cutting limits and park management parameters by consensus (Curran 2019; *Haida Gwaii Reconciliation Act* 2010). As a direct and ongoing governance body, the Haida Nation has significantly more influence on natural resource development and land use planning than do Indigenous nations operating within either the aboriginal rights framework of consultation or reacting via the *EA Act* or BC *DRIPA* in response to project proposals. This example underscores the possibility of joint Indigenous-state decision-making structures embedded in state law that flow from the constitutional affirmation of aboriginal rights and superseded everyday statutory processes.

## The Mining Law Regime in Sweden/Norway and the Sami

### Context: Mine Developments and Sami Rights

In large parts of Sápmi, the traditional territory of the Indigenous Sami people,^13^ mineral deposits are abundant. Both Sweden and Norway have a longstanding history of mining activities; mining commenced on a larger scale towards the end of the 16th century (Ojala & Nordin 2015: 12; Strøm Bull K (1997): 362–3). Today, Sweden, in particular, is an important producer within the European Union of ore and metals, and is, for instance, Europe’s top producer of iron ore (SOU 2018:59, 35)^14^ Sweden and Norway’s Minerals Strategies (Government Offices of Sweden 2013; Norwegian Ministry of Trade, Industry and Fisheries 2013) emphasize the goal of promoting growth within the mining and minerals industry to respond to increasing global and regional demand as well as to support the transition to green energy supplies in general.

The actual and anticipated increase in prospecting activities and expansion of mines is especially controversial in traditional Sami territories (Raitio et al. 2020; Beland Lindahl et al. 2018; Nygaard 2016; Koivurova et al. 2015). The geographical area of Sápmi roughly corresponds with the traditional Sami reindeer herding areas where Sami need vast grazing areas on state-owned and privately owned lands (Allard 2011: 163-65). In Swedish and Norwegian Sápmi Sami herd management is based on reindeer grazing in natural and open “pastures” across a variety of ecosystems. This means that their land-use commonly collides with other industries such as mining, forestry, wind farms, tourism, and infrastructural developments. Reindeer herding, managed in specific reindeer herding communities with fairly defined geographical boundaries, is a collective nomadic livelihood and cultural practice and is still a foundation of Sami culture; it carries traditions, knowledge, and language from one Sami generation into the next (Raitio et al. 2020: 3). Sami coastal and fjord fishing, especially in northern Norway, is also an important activity that uses Sami traditional knowledge (e.g., Kalak and Johansen 2020).

Like the Indigenous communities in BC, the Sami have been subjected to colonial marginalization and assimilation policies that have depleted their traditional social, economic, cultural, and political institutions, including their customs and customary laws (Andresen et al. 2021; Minde 2005; Lundmark 2002). The closest thing to a historical treaty that exists, upholding Sami customary rights, is the Lapp Codicil, an appendix to a border treaty from 1751, that aimed to finalize the Norwegian-Swedish national border in the far north (Pedersen 2008). It is still formally applicable.

Legal developments in relation to the Sami an Indigenous people, in both Sweden and Norway, correspond largely to international human rights treaties, and especially so with respect to the state duty to consult the Sami (Allard 2018). Among the three Nordic countries, only Norway has ratified the International Labour Organization (ILO) Convention 169^15^, a document that upholds the rights of Indigenous and tribal peoples. The matter of Sami land rights has been particularly difficult for Sweden and Finland, thus stalling potential ratification; it is, in particular, the unresolved Sami land rights in the northern territory of the two countries that have caused national debates as the region is rich of natural resources.^16^ The ILO Convention 169 is, however, not directly incorporated in Norwegian law, but partially acknowledged via provisions in certain legislation (Funderud Skogvang 2017: 121–3), such as the *Minerals Act, 2009* section 6. It states that “[t]he Act shall be applied in accordance with the rules of international law relating to indigenous peoples and minorities.” So far, UNDRIP has not played any significant role in litigations or legislative processes in Sweden or Norway to the benefit of the Sami.

Another common feature, however, is that Sweden and Norway’s constitutions have been and continue to be rather weak, especially in comparison to common law states such as Canada. Constitutional provisions are seldom evoked before courts and, as a result, usually play only a minor role in specific disputes and legal application. In Sami-related cases, however, it is more common to rely on constitutional provisions aimed at protecting Sami culture and/or property. In 1988 the so-called Sami clause was added to Norway’s Constitution of 1814; one of the main purposes was to reconcile past grievances and assimilation policies (NOU 1984:18, 432).^17^ Section 108 states: “It is the responsibility of the authorities of the State to create conditions enabling the Sami people to preserve and develop its language, culture, and way of life.” As a result, the Constitution places legal responsibility on the public authorities of Norway, and the constitutional provision serves as an aid in the interpretation of ordinary legislation (Funderud Skogvang 2017: 188). Comparatively, the Swedish constitutional provision in the *Instrument of Government, 1974* is weaker and states (ch. 1s. 2 para. 6): “The opportunities of the Sami people and ethnic, linguistic and religious minorities to preserve and develop a cultural and social life of their own shall be promoted”. Indeed, it is hardly ever referred to in litigations.

In Sweden and Norway the right to herd reindeer is exclusively and historically codified into specific Reindeer Herding Acts that today award the same constitutional protection against infringements as ownership (i.e., a right to be compensated) (Allard 2011: 165–66). In Norway, the legal bases for the State’s duty to consult Sami are now enshrined in legislation. A bill (Prop. L 86 (2020–2021))^18^ was recently approved by the Norwegian Parliament, in June 2021, that essentially codifies the previous legal situation and builds upon agreement between the state government and the Sami Parliament^19^ of Norway (Allard 2018). Sweden lacks any provisions that impose a duty of consultation on the Swedish State (ibid). The matter, however, has been investigated and a law reform proposal exists (Prop. 2020/21:64) that the Swedish Parliament will consider later in 2021.

### Mining Permitting

The Norwegian *Minerals Act, 2009*, shows some similarities with the Canadian free entry principles, but with the addition of specific provisions to protect Sami interests in the county of Finnmark—a core Sami area in the far north of Norway. The Norwegian *Minerals Act, 2009* differentiates “state minerals” from “landowner’s minerals”, where the exploration and exploitation of the latter is subject to agreements between the proponent and the landowner (ss. 7, 11, 28). This is alien to Swedish mineral legislation.

Instead, Sweden’s *Minerals Act, 1991* rests on the idea that the landowner owns the minerals (cf. ch. 5s. 2 of the Act), in theory at least (Bäckström 2015: 48), and hence operates under a private mineral rights regime. However, this system lacks practical relevance because any mining company can be given rights to private property for prospecting activities and minerals via permits if the agency approves an application (*Minerals Act* ch. 2s. 2 and ch. 4s. 2). The *Minerals Act, 1991* is applicable only for explicitly listed “concession minerals” of industrial use and economic value (ch. 1s. 1). Hence, an approved mining permit establishes an exclusive right for the proponent to dispose of particular minerals within a designated permit area irrespective of whether the minerals are located on public or private land (ch. 5s. 1).

Whereas Swedish mineral legislation is silent on any specific provisions to protect Sami rights and culture, Norway’s *Minerals Act* from 2009 does incorporate some level of protection for Sami interests. Within the purpose of the Act—to promote and ensure socially responsible management of minerals in accordance with sustainable development (s. 1)—Sami interests are among the mentioned interests and must be specifically addressed and balanced. Following section 2, in assessing permits under the Act, “the foundation of Sami culture, livelihoods, and social life” shall be taken into consideration (point b). This provision is generally applicable across Norway, meaning that there is a basic consideration of Sami interests and livelihoods in mine developments. Specifically for applications in Finnmark, section 17 states that:

A special permit *may be refused* if granting the application would be contrary to Sami interests. In the assessment, special consideration shall be given to the interests of Sami culture, reindeer management, commercial activity, and social life. If the application is granted, conditions may be imposed to safeguard these interests. (Emphasis added)

This safeguarding applies for exploration as well as extraction, but only in Finnmark (cf. s. 43). It should be noted that the Norwegian Sami Parliament and Sami organizations did not approve of the 2009 Act; the state conducted several consultation rounds in the law-making process, on the basis of the consultation agreement, and the Sami maintained that the Act was not in line with international law and did not adequately protect Sami outside Finnmark (Report 2018: 37).

Sweden’s *Minerals Act* does not single out the Sami as an Indigenous people. However, with respect to the assessment of the mining permit, provisions in the *Environmental Code, 1998* (chs. 3-4) do mention Sami reindeer herding. These provisions are a vital part of the assessment and the trade-offs being made. Balancing is required, where values in specifically mentioned “areas of national interest” for both conservation and exploitation are assessed simultaneously, essentially examining whether the interest of minerals and mining can co-exist with reindeer herding in the designated area. Under the specific provision reindeer herding is regarded as a *public interest* aimed to safeguard an important Sami cultural activity (ch. 3s. 5). This trade-off normally ends with concluding that the co-existence of Sami rights and mining is possible (Raitio et al. 2020).

A common denominator of the Swedish and Norwegian mining law systems is that an agency is authorized to make decisions about whether a mine is approved under the *Minerals Acts*, after consulting with municipalities and other relevant agencies. In the case of contradictory views, the decision is elevated to the government (Sweden: Ministry of Enterprise and Innovation/Norway: Ministry of Trade, Industry and Fisheries). Another similarity is that apart from authorizations under the mining legislation, several other permits are required for mine development, notably environmental permits and permits for buildings, road construction, and other activities. The key threshold for a successful mining permitting process in Norway is the early zoning plan with a comprehensive impact assessment (IA) under the *Planning and Building Act, 2008* (ss. 4-2 and 12-1), where the municipality may stop mining projects at an early stage, essentially through zoning (by not designating the area for industrial purposes). The Norwegian IA has a broader scope than just impacts on the environment, a feature that has existed since the early 1980s (Tesli and Lund-Iversen 2014: 83–4). A second threshold is the final mining permit approval under the *Minerals Act* (s. 43) (Report 2018: 55–58).

Under Swedish law the key threshold is the mining permit under the *Minerals Act* (ch. 4s. 2); if approved the responsible agency usually issues the environmental permit (Raitio et al. 2020). In the Swedish system two environmental impact assessments (EIAs) are conducted as part of the applications for the mining permit as well as the environmental permit. The scope of the EIA is narrow and allows consideration of *environmental* impacts only (Raitio et al. 2020; Pölönen et al. 2020). Currently, due to the political delicacy of a handful of applications for mining permits, the Swedish Government has stalled the assessment of those applications. Sami rights are a key consideration in these cases (Raitio et al. 2020). Conversely, in 2018 the Norwegian Government approved a new mine called Nussir, located in the county of Finnmark, where safeguarding Sami interests is enhanced (Allard 2021). One of the conditions of the mining permit is, for instance, that the mining activities shall completely stop for almost two months each year during the sensitive period for reindeer calving and migration (Norwegian government decision 2018). In both Sweden and Norway, legislation secures compensation to Sami reindeer herding communities, land owners, and other right-holders for adverse effects of an approved mine project.

In summary, two things are evident from the noticeably different Swedish and Norwegian mining legislative regimes. The first is that only Norway’s *Minerals Act* explicitly includes provisions that consider Sami rights and interests. While the Norwegian legal geography is limited to Finnmark regarding the specific provisions protecting Sami interests, there is the opportunity to expand those provisions outside Finnmark.^20^ Second, there is a pressing need for a comprehensive overhaul of Swedish mining legislation as well as other sector-specific legislation to secure adequate protection of Sami rights and interests.^21^ Given the more extensive engagement between the state and Indigenous people in BC, what could the two Nordic states learn from the legal situation and possibilities for engagement in BC?

## Opportunities for Indigenous Participation and Engagement

In the context of mining and engagement between Indigenous peoples and state governments, several features of the Canadian constitutional and BC administrative law regimes stand out as offering opportunities for other states as they turn to more meaningfully engaging Indigenous peoples in decision-making about mining activities. The first is the overarching purpose of reconciliation that underpins the constitutional acknowledgement of aboriginal and treaty rights. The second aspect is the new attempts to apply UNDRIP within state law, specifically to align state laws with UNDRIP and to develop consent-based regulatory processes. The third feature is the spectrum of consultation approaches depending on the scale of a proposed activity and the depth of Indigenous interests in a particular location or ecological process. Finally, there is considerable untapped potential for the EA process to be conducted in partnership with or led by Indigenous organizations, including having their costs covered, that offers opportunities for operationalizing reconciliation, consent-based processes, and more respectful relationships between Indigenous communities and the state.

A principle in Canada’s policy and constitutional environment relating to Indigenous peoples is reconciliation. The underlying purpose of reconciliation is to find resolution to a problem of some kind, normally a dispute, and to reconcile something is thus to find a way to make two or more ideas or situations agree with each other, when they actually seem to be in opposition.^22^ New legislation in BC, namely the *EA Act* and BC *DRIPA*, both refer to reconciliation and to UNDRIP. It is notable that through BC *DRIPA* BC was the first province in Canada to bring the international human rights standard of UNDRIP into provincial law (Office of the Premier 2019). Both an overarching ideal, such as reconciliation, and implementing UNDRIP into domestic laws in Sweden and Norway would be important motivations for recognizing Sami rights in general and particularly with respect to mine development. Sweden lacks altogether an ideal or political goal relating to the Sami as an Indigenous people (Allard 2006: 500–1) whereas Norway, in various documents from 2000 onwards has declared that it is founded on the territory of two peoples: the Norwegians and the Sami (Gauslaa 2007: 155). This basic idea forms the foundation of Norway’s Sami policy today and supports the perception of a partnership between the Sami and the State. A move towards reconciliation, or a similar notion, would not be as far-fetched for Norwegian law and policy as it would for the Swedish. The implementation of UNDRIP into domestic law would mean developing consent-based decision-making processes with respect to mine development processes and decisions. The state duty to consult the Sami works in tandem here; the bigger the negative effects on reindeer herding and other Sami livelihoods of a proposed project, the greater the need for continuous, in-depth consultations and negotiations with the Sami.

However states choose to begin implementing UNDRIP, Canada, and BC in particular, has considerable experience engaging in a spectrum of consultation approaches. How extensive consultation and engagement is for any proposed activities depends on the scale of the activity—in size, impact on aboriginal or treaty rights, and duration—as well as the depth of Indigenous interests in a particular location or ecological process. As a first step, Sweden and Norway could affirm Sami rights to make decisions on their traditional lands and commit to creating ongoing processes of engagement with Sami communities. Established as engagement or decision-making tables and based on Sami-state protocol agreements, these engagement venues could consider all manner of proposed activities, as well as work towards long-term land and water use plans that create common agreement about what will occur across Sami traditional territory well into the future (BC Mining Law Reform Network, 2020).

Finally, BC’s system for EA of proposed mines (as well as other environmental hazardous activities) is notable because it: (i) is run by an independent (at least in structure) EA Office that is responsible for administering the EA; (ii) offers a system of choice for Indigenous communities either to create their own Indigenous-led assessment, take part in the state-led process, or not engage at all; and, (iii) provides financial compensation for the work done by Indigenous communities with the assessments. All three aspects of BC’s EA system would heighten the role of affected Sami reindeer herding communities in Sweden and Norway in relation to such assessment procedures. They would at least be provided with compensation for their work with EA’s even though it is important to note that payment for EA work is not compensation for impacts on Sami reindeer herding communities. They commonly argue that state and proponent knowledge of their land use is too shallow (Kløcker-Larsen et al. 2018).

## Conclusion

The primary purpose of this article is to focus on the areas of strength in state legal processes that enable engagement with Indigenous people in BC in relation to mining activities, and to apply a comparative lens to the laws in Sweden and Norway with respect to mine developments and Sami rights. We asked what the BC regime could offer to the two Nordic countries in terms of creating processes for engaging with Sami and considering their interests in decisions about mining. What is evident from this short comparison is that the combination of mine developments and securing Indigenous authority and influence over land uses in their traditional territories is extremely challenging for all three state governments, and current mining legislation displays a clear prioritization of state interests, historical colonial legacies and pro-development tendencies. The clear commitment of all states to boost mineral exploitation, especially in the context of “critical minerals” for the energy transition, adds an extra layer of complexity to resource developments in traditional Indigenous territories.

Before addressing a few comparative points, we first summarize highlights from the analysis of the BC legislation. Mindful of the shortcomings of laws related to mine development in BC—especially with respect to the first phase of the approval process where the mineral tenure regime permits staking a mineral claim across much of BC without considering Indigenous laws and processes—recent legal developments hold potential for significantly increasing the role of affected Indigenous communities, at least in parts of the permitting process. BC *DRIPA* is a short law that aims to align BC law with UNDRIP within the framework of reconciliation. This Act requires the BC Government to review laws pertaining to all phases of the permitting process and confront the critiques set out above.

Equally important is the *EA Act*, under which one of the key approvals is the EA certificate that is required to continue with a major mine. Throughout the *EA Act* are opportunities for Indigenous communities to determine their level of engagement and provide or withhold consent in consensus-like procedures. The *EA Act* also contemplates providing compensation for the participation of Indigenous communities proportionate to their work, and permits a broad assessment scope—the economic, social, cultural, and health effects of assessed projects—that reflects Indigenous peoples’ lived experience. Finally, the quasi-independent EA Office is responsible for seeking consensus with Indigenous nations and in preparing the draft assessment report which, in structure, is more impartial compared to the Swedish and Norwegian regimes where the proponent has the sole responsibility for the assessment.

This leads us to the key features of our comparison. Sweden and Norway, but especially Sweden, will require significant legal reform to achieve the standards established by UNDRIP, the concept of reconciliation, and to move towards the approaches to Indigenous engagement in mine developments set out by current BC legislation. The overall ideal of reconciliation offers a platform for engagement and negotiation unparalleled in Sweden or Norway, although Norwegian politics actually recognize that Norway is founded on the territory of the two peoples, thereby fostering nation-to-nation engagement. The notion of reconciliation (or similar concepts) in Sweden and Norway, even if only a political goal, would provide a clearer direction for mining and environmental laws, including assessment procedures. However, for both countries international law plays a vital role as the foundation for special rights for the Sami in their capacity as an Indigenous people, including the state duty to consult the Sami. Domestic legal developments related to reconciliation, such as those evidenced in Canada and backed by its highest court, cannot take place in these Nordic legal systems with their weaker court structures (Allard 2006: 500–1, 50–7-8).

A comparative insight that offers greater potential for upholding Indigenous authority, therefore, relates to UNDRIP. BC has enabled consent-based processes for environmental assessments, which also could be extended to mining permitting processes (or other land and natural resource decisions). Such an approach would greatly benefit processes in both Sweden and Norway. BC law also offers a spectrum of consultation and engagement approaches from which the two Nordic countries could learn. Authority to engage with Indigenous communities flows from the constitutional duty to consult and accommodate, specific legislation (such as the *EA Act*), and government-to-government agreements that specify consultation processes in relation to particular types of land-use decisions. Finally, the structure and potential for improving (environmental) impact assessments for mine developments are significant. In BC, the EA process can be conducted in partnership with or led by Indigenous organizations, with some compensation for costs incurred for the EA. This type of assessment process would foster more respectful relationships between Sami communities, mining companies, and the state, as well as increase respect for Sami traditional knowledge.

Overall, the new BC legislation offers procedural means to support Indigenous influence and engagement in the decision-making processes relating to mine development. At the end of the day, however, the ultimate power rests with the state governments, both in permitting mineral extraction throughout Indigenous lands and in making the final decision about mining activities. Therefore, substantive provisions safeguarding Indigenous communities’ livelihoods and interests are fundamentally required to meaningfully implement UNDRIP.

## Footnotes

1 In this article the term “Indigenous communities”, the preferred term, refers to self-identified groups of Indigenous peoples. The term “First Nation”—as distinct from Métis and Inuit—has become the usual way of referring to groups known as Indian Bands under the federal *Indian Act* and are typically the political organization through which Indigenous communities interact with the state. Under section 35 of the *Canadian Constitution Act, 1982*, Indians, Métis, and Inuit are all included in the term “aboriginal peoples.” It is more common in Canada to refer to Indigenous peoples (rather than Aboriginal), which is the term used in this article. Relatedly, there are many different types of governments when discussing Indigenous and non-Indigenous legal orders. To differentiate and in recognition that settler or colonial governments have status in international law, we use the term “state” to denote national and sub-national settler governments.

2 The focus on Sweden and Norway (and not Finland or Russia) is explained by the two research projects to which this article is connected (see Acknowledgements).

3 BC has a vast landmass, 944,735 square kilometers in size (Robinson et al. 2020), thus larger than many countries in the world.

4 One example is the Britannia copper mine (1905–1974) on Howe Sound, 40 kilometers north of Vancouver and within the unceded territory of the Squamish Nation. It is known as one of the largest point sources of metal pollution in North America (Province of BC, no date-a).

5 The Xeni Gwet’in First Nations, as also represented by the Tsilhqot’in National Government, in the central part of BC is the only First Nation that has proven aboriginal title through Canadian courts. The duty to consult veers towards a consent-like framework for those Nations. Tsilhqot’in Nation, 2014.

6 Hereinafter named the BC *DRIPA*.

7 An Indigenous governing body is defined (s. 1) as an entity that is authorized to act on behalf of Indigenous peoples that hold rights recognized and affirmed by section 35 of the *Constitution Act, 1982*.

8 The Auditor General and the Office of the Auditor General is independent of government and is tasked with conducting audits, reporting on how well the government is managing its responsibilities and resources, and making recommendations for continuous improvement. The Auditor General reports directly to the Legislative Assembly. See https://www.bcauditor.com/about-us/who-we-are (Accessed 9 March 2021).

9 Mineral title means a mineral claim or a lease, see *Mineral Tenure Act* s. 1.

10 They liaison with natural resource agencies including the Ministry of Energy, Mines and Low Carbon Innovation, the Ministry of Environment and Climate Change Strategy, and the Ministry of Forests, Lands, Natural Resource Operations, and Rural Development. An advisory Project Board, consisting of senior-level leaders from the Province’s natural resource agencies, is also linked to the Major Mines Office (BC Major Mines Office, no date).

11 Expansion of an existing mineral mine triggers an EA where the production level meets the 75,000 tonnes per year minimum and additional land disturbance of at least 50 percent of the existing permitted site disturbance. See *Reviewable Projects Regulation* s. 10(1).

12 Note that a mining project cannot proceed on treaty land if the treaty requires consent of the Indigenous nation or in an area that requires consent and is identified by regulation (s. 7).

13 Sápmi stretches over Norway, Sweden, Finland, and Kola Peninsula in Russia. For a map see http://www.samer.se/1002 (Accessed 19 March 2021).

14 “SOU” is a report by a Swedish Government Commission that forms part of the preparatory works, a document that continues to be an important source of law among the Nordic countries”.

15 The ILO Convention Concerning Indigenous and Tribal Peoples in Independent Countries, 1989 (No. 169).

16 Finland was part of the Swedish Kingdom for 600 years, before 1809, with the same laws and, therefore, the legal-historical situation is very similar (Allard 2015: 50–51, 57).

17 “NOU” is a report by a Norwegian Government Commission that forms part of the preparatory works, a document that continues to be an important source of law among the Nordic countries.

18 A “Prop” is a government bill and is an important legal source in the Norwegian and Swedish legal systems that forms part of the preparatory works.

19 The Sami Parliament, to some extent offering Sami self-determination, was established by an Act of Parliament and its mandate is regulated by the same act. Sweden has also established a Sami Parliament, by an Act of Parliament, but with somewhat different powers.

20 This is actually being assessed by a public commission tasked to suggest amendments to the Act, and report back by December 2021. See news from Norwegian Government: https://www.regjeringen.no/no/aktuelt/vil-ha-lonnsom-og-barekraftig-mineralvirksomhet/id2715431/ (Accessed 16 March 2021).

21 A public commission is established (March 2021) but relates only to innovation critical minerals and materials. The Sami or reindeer herding is not even mentioned and thus not part of the assessment. See Dir. 2021:16.

22 Cf. the Oxford Advanced Learners Dictionary.
